# Serological Surveillance of the H1N1 and H3N2 Swine Influenza A Virus in Chinese Swine between 2016 and 2021

**DOI:** 10.1155/2022/5833769

**Published:** 2022-04-28

**Authors:** Yuzhong Zhao, Lebin Han, Ting Chen, Haotian Sang, Guofei Ding, Yingchao Li, Bin Wang, Liting Qin, Sidang Liu, Yanmeng Hou, Yihong Xiao

**Affiliations:** ^1^Department of Fundamental Veterinary Medicine, College of Animal Science and Veterinary Medicine, Shandong Agricultural University, 61 Daizong Street, Tai'an 271018, China; ^2^Shandong Provincial Key Laboratory of Animal Biotechnology and Disease Control and Prevention, Shandong Agricultural University, Tai'an 271018, China; ^3^Shandong New Hope Liuhe Group Co., Ltd., Qingdao 266100, China; ^4^Qingdao Jiazhi Biotechnology Co., Ltd., China

## Abstract

**Background:**

Swine influenza A virus (IAV-S) is a common cause of respiratory disease in pigs and poses a major public health threat. However, little attention and funding have been given to such studies. The aim of this study was to assess the prevalence of the Eurasian avian-like H1N1 (EA H1N1), 2009 pandemic H1N1 (pdm/09 H1N1), and H3N2 subtype antibodies in unvaccinated swine populations through serological investigations. Such data are helpful in understanding the prevalence of the IAV-S.

**Methods:**

A total of 40,343 serum samples from 17 regions in China were examined using hemagglutination inhibition (HI) tests against EA H1N1, pdm/09 H1N1, and H3N2 IAV-S from 2016 to 2021. The results were analyzed based on a reginal distribution, seasonal distribution, and in different breeding stages.

**Results:**

A total of 19,682 serum samples out of the 40,343 were positive for IAV-S (48.79%). The positivity rates to the EA H1N1 subtype, pdm/09 H1N1 subtype, and H3N2 subtype were 24.75% (9,986/40,343), 7.94% (3,205/40,343), and 0.06% (24/40,343), respectively. The occurrences of coinfections from two or more subtypes were also detected. In general, the positivity rates of serum samples were related to the regional distribution and feeding stages.

**Conclusions:**

The results of this study showed that the anti-EA H1N1 subtype and pdm/09 H1N1 subtype antibodies were readily detected in swine serum samples. The EA H1N1 subtype has become dominant in the pig population. The occurrences of coinfections from two or more subtypes afforded opportunities for their reassortment to produce new viruses. Our findings emphasized the need for continuous surveillance of influenza viruses.

## 1. Background

Swine influenza A virus (IAV-S) is an acute respiratory viral disease mainly caused by influenza A viruses (IAV) of the Orthomyxovirus family [1, 2]. The symptoms of viral infection are fever, anorexia, tachypnea, dyspnea, and coughing [3]. The morbidity rate of IAV-S can reach 100%, whereas the mortality rate can be as low as 1% [4]. However, when combined with other respiratory pathogens, the disease will be aggravated and result in high mobility and mortality.

Swine play an important role in the ecology of IAV. The cells of the swine respiratory tract express two receptor types, including human (N-acetylneuraminic acid-a2,6-galactose) receptors and avian (N-acetylneuraminic acid-a2,3-galactose) receptors [5, 6]. Thus, swine are susceptible hosts for avian, swine, and human influenza viruses, and are considered as “mixing vessels” for the generation of pandemic-potential influenza viruses through reassortment [7, 8].

Diverse influenza virus lineages are present in the pig population, including the classic swine H1N1 (CS H1N1) virus lineage, Eurasian avian-like H1N1 (EA H1N1) virus lineage, 2009 pandemic H1N1 (pdm/09 H1N1) virus lineage, North American triple-reassortant virus, and H3N2 virus lineage [9, 10]. Despite collaborations on influenza research between international organizations such as the World Organization for Animal Health, Food and Agriculture Organization of the United Nations, and the World Health Organization being established to share information and resources [11], relatively little is known regarding the IAV-S circulating in swine in China and around the world. Surveillance of IAV-S circulating in pigs and other nonhuman mammals has been chronically underfunded or nonexistent in many areas of the world, even after the emergence of the pandemic H1N1 in 2009 [12]. The paucity of data continues despite awareness of its threat to humans and other animals [11].

Several diagnostic tests for influenza, including viral culture, serology, rapid antigen testing, RT-PCR, immunofluorescence assays, and rapid molecular assays are currently available. Although routine serological testing for influenza requires paired acute and convalescent sera, it does not provide results that assist with clinical decision-making (Centers for Disease Control and Prevention (2017), clinical signs, and symptoms of influenza. https://www.cdc.gov/flu/professionals/acip/clinical.htm.(accessed 20 Nov., 2017)). However, serological tests are advantageous in retrospective studies of virus epidemics. In the present study, serum samples collected from pigs not vaccinated against any subtype of IAV-S were detected for the presence of EA H1N1, pdm/09 H1N1, and H3N2 subtypes in China from 2016 to 2021, as those data can provide useful information on the epidemiology of IAV-S.

## 2. Methods

### 2.1. Ethics Statement

This study was approved by the Ethics Committee of Shandong Agricultural University (Tai'an, China). The project identification code is 2016-004, which was approved on 25 April 2016 (25/04/2016). All serum samples were collected under the guidelines of the Animal Care and Use Committee of Shandong Agricultural University.

### 2.2. Serum Samples

**Figure 1:**
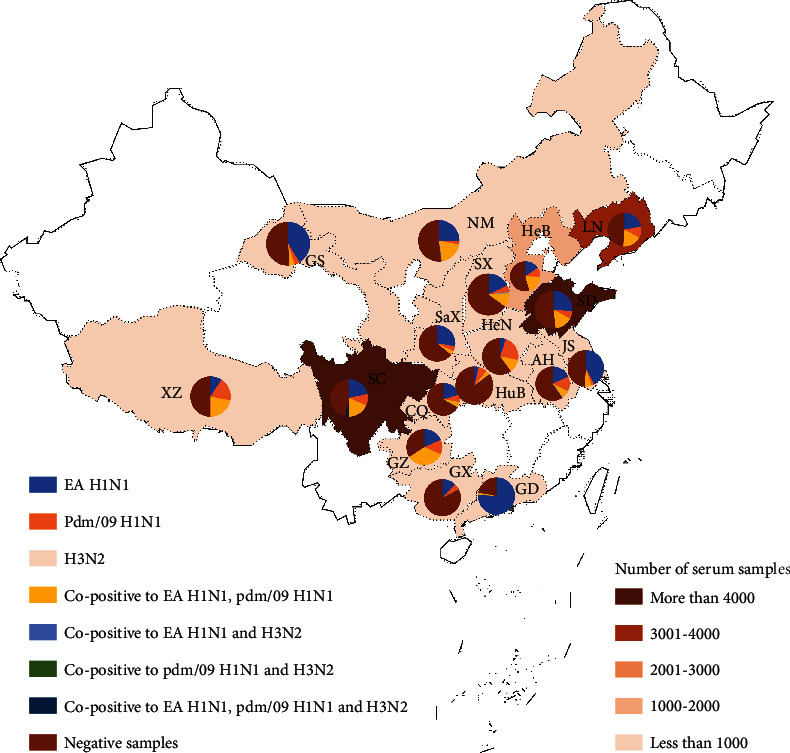
2016-2021 IAV-S serological test results in different provinces of mainland. The number of serum in different provinces was counted, and the relative proportion of various subtypes in different provinces was illustrated by pie chart. Different colors represent different provinces and subtypes. Data for 17 provinces of China are presented as follows. LN: Liaoning *n* = 3584; NM: Neimenggu *n* = 314; HeB: Hebei *n* = 1082; SX: Shanxi *n* = 157; SD: Shandong *n* = 23485; GS: Gansu *n* = 100; SaX: Shaanxi *n* = 134; HuB: Hubei *n* = 27; HeN: Henan *n* = 100; AH: Anhui *n* = 654; JS: Jiangsu *n* = 482; SC: Sichuan *n* = 9300; CQ: Chongqing *n* = 384; GZ: Guihou *n* = 38; GX: Guangxi *n* = 420; GD: Guangdong *n* = 50; XZ: Xizang *n* = 32.

From October 2016 to August 2021, a total of 40,343 serum samples from pigs in 17 areas (Guangdong, Guizhou, Sichuan, Jiangsu, Liaoning, Xizang, Neimenggu, Gansu, Hebei, Shandong, Henan, Anhui, Shanxi, Shaanxi, Chongqing, Guangxi, and Hubei provinces) of China were collected. The sizes of pig farms where samples were collected varied from large-scale operations (>7,000 swine) to backyard farms (<400 swine). All samples were from pigs that were not vaccinated against any subtype of IAV-S. All pigs were healthy and did not have any clinical signs of influenza, such as coughing, sneezing, and nasal mucus (Figure 1). Samples from pigs at all life stages, including piglets, nursery pigs, fattening pigs, gilts, sows, and boars, were collected from each location. Blood samples were collected by jugular puncture and immediately transported to the laboratory. Samples were placed at room temperature and centrifuged at 5,000 × g for 5 min after clot formation (Multifuge X1 Pro, Thermo Scientific). Serum samples were stored at -20°C until analysis.

### 2.3. Antigens

Antigens of H1N1 (EA H1N1 and pdm/09 H1N1 subtypes) and H3N2 subtypes were provided by Harbin Veterinary Research Institute, Chinese Academy of Agriculture Sciences (Harbin, China).

### 2.4. Hemagglutination Inhibition (HI) Tests

Before the HI tests were performed, all serum samples were treated using the Trypsin-Heat-Periodate method (General Administration of Quality Supervision, Inspection, and Quarantine of the People's Republic of China, Standardization Administration GB/T 27535-2011 Detection method of hemagglutination inhibition antibody against swine influenza [S]. Beijing: Standards Press of China, 2012. (in Chinese)) to remove nonspecific hemagglutinating inhibitors. In brief, 15 *μ*L of trypsin (0.8% *W*/*V*) was added to 30 *μ*L of serum, and the mixture was incubated for 30 min at 56°C. The samples were then cooled to room temperature, and immediately after, 90 *μ*L of potassium periodate (0.01 mol/L) was added. The mixture was then incubated at room temperature for 15 min. Then, 90 *μ*L of glycerin (1%) was added, and the mixture was incubated at room temperature for 15 min. Finally, 75 *μ*L of phosphate-buffered saline was added, and the mixture was stored at 4°C.

The prepared serum samples (30 *μ*L) were mixed with four hemagglutinin units of virus (30 *μ*L) in 96-well plates and cultured at room temperature for 15 min. A volume of 30 *μ*L of chicken erythrocytes (1%) was added to each well, mixed, and then cultured at room temperature for 15 min. In each test conducted, the serum (standard positive serum) against EA H1N1, pdm/09 H1N1, and H3N2 IAV-S were used as positive controls, and SPF chicken serum (standard negative serum) was used for negative controls. HI antibody titers ≥1: 10 were defined as serologically positive.

## 3. Results

All serum samples were tested for antibodies specific to EA H1N1, pdm/09 H1N1, and H3N2 subtype viral antigens using the HI test. Of the 40,343 pig serum samples tested, 19,682 (48.79%) were positive. Among which, the highest positivity rate was for EA H1N1 (9,986; 24.75%), followed by pdm/09 H1N1 (3,205; 7.94%). Only 24 samples were positive for H3N2 (0.06%). Copositive samples were also analyzed, with 6389 being positive for EA H1N1 and pdm/09 H1N1 (15.84%). A total of 37 samples were copositive for H3N2 and EA H1N1 (0.09%), while 18 samples were copositive for pdm/09 H1N1 (0.04%). A total of 23 samples were positive for all antigens, including those from EA H1N1, pdm/09 H1N1, and H3N2 (0.06%; Table 1). Thus, the results showed that positivity to the H1N1 subtype was dominant. There were also cases of mixed infections involving two or three subtypes.

### 3.1. Regional Distribution of IAV-S-Positive Samples to China from 2016 to 2021

All samples were collected from 17 regions of China, and the positivity rates were different between each region (Figure 1). Guangdong province had the highest positivity rate of 78.00%, followed by Guizhou (65.79%), Sichuan (52.52%), Jiangsu (51.04%), Liaoning (51.03%), Xizang (50.00%), Neimenggu (49.36%), Gansu (49.00%), Hebei (45.73%), Shandong (48.63%), Henan (40.00%), Anhui (40.83%), Shanxi (36.30%), Shaanxi (35.82%), Chongqing (33.59%), Guangxi (16.90%), and Hubei (14.80%) provinces. Thus, the results indicated that the positivity rates were different based on the reginal distribution. The positivity rates of samples from regions of South China (including Guangdong, Guizhou, and Jiangsu) were higher than those from other regions. The overall seroprevalence of the EA H1N1 subtype was higher than that of the pdm/09 H1N1 or H3N2 subtypes. The results showed that antibodies against EA H1N1 were most dominant in swine herds of China, thus, indicating the prevalence of the EA H1N1 subtype. With the exception of Xizang, Henan, and Hubei provinces, the positivity rates of the EA H1N1 subtype were higher than those of the other two subtypes in all other province (Figure 1). In Guangdong province, 38 of 50 samples were positive for the EA H1N1 subtype, and one sample was copositive for the EA H1N1 and pdm/09 H1N1 subtypes (78.00%). EA H1N1 positivity rates were comparably high in Jiangsu and Gansu provinces with 42.32% and 41.00%, respectively. These results indicated that the positivity rates of the EA H1N1 subtype were higher in South China (Guangdong, Jiangsu) than in other regions. Similar positivity rates to the EA H1N1 subtype were also detected in Sichuan, Anhui, Liaoning, Hebei, Shaanxi, Guizhou, Neimenggu, Shanxi, and Guangxi, ranging from 12.14% to 27.61%. In Xizang, Henan, and Hubei provinces, the positivity rates for EA H1N1 ranged from 3.7% to 9.38, which were lower than those of the pdm/09 H1N1 subtype. For the pdm/09 H1N1 subtype, the highest positivity rate (23.00%) was detected in Henan province, followed by that in Xizang, Anhui, Guizhou, and Liaoning with positivity rates of 18.75%, 13.76%, 13.16%, and 10.88%, respectively. In the other 10 provinces, overall positivity rates of less than 10% were detected. In Guangdong province, no samples were positive for the pdm/09 H1N1 subtype. Moreover, only a few samples were positive for the H3N2 subtype in Shandong, Sichuan, Anhui, Chongqing, and Shanxi provinces with rates ranging 0.05% to 1.27%. Copositivity for the EA H1N1 and pdm/09 H1N1 subtypes was observed in all 17 provinces. The highest positivity rate was detected in Guizhou province (34.21%). Similar positivity rates of 18.66%, 18.00%, 20.43%, 21.88%, and 20.06% were found in Sichuan, Liaoning, Hebei, Xizang, and Neimenggu provinces, respectively. Copositivity rates for the EA H1N1 and pdm/09 H1N1 subtypes of about 10% were also detected in Shandong (15.29%), Henan (12.00%), and Shanxi (10.80%) provinces. Less than 10% copositivity rates for the EA H1N1 and pdm/09 H1N1 subtypes were detected in other provinces. Only one sample in Guangxi province was positive for the EA H1N1 and pdm/09 H1N1 subtypes, resulting in a positivity rate of 0.24%. A few samples from Shandong, Sichuan, Anhui, Neimenggu, and Shanxi provinces were positive for the EA H1N1 and H3N2 subtypes with rates of 0.11%, 0.08%, 0.31%, 0.64%, and 0.06%, respectively. Samples copositive for the pdm/09 H1N1 and H3N2 subtypes were only detected in Shandong, Guangxi, and Shanxi provinces, with rates of 0.07%, 0.24%, and 0.06%, respectively. Triple positivity to EA H1N1, pdm/09 H1N1, and H3N2 subtypes was also detected in Shandong, Sichuan, Anhui, and Chongqing provinces. All copositive groups were detected in Shandong province, which may be due to the largest number of samples being from that province.

### 3.2. Seasonal Distribution of IAV-S-Positive Samples in China from 2016 to 2021

The positivity rates of all samples were analyzed yearly and seasonally. The average annual seropositivity rates from 2016 to 2021 were 45.41%, 50.01%, 66.86%, 50.53%, 45.07%, and 45.73%, respectively. Among the years, the highest seropositivity rate was detected in 2019, while antibodies against EA H1N1, pdm/09 H1N1, and H3N2 subtypes were detectable in each year from 2016 to 2021.

**Figure 2:**
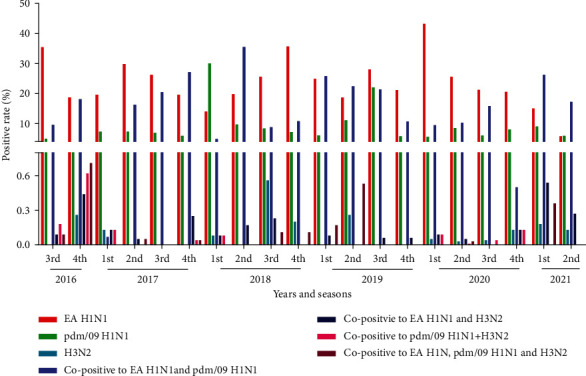
Serological survey of the different dates of IAV-S in the swine population in China, 2016-2021.

For the EA H1N1 subtype, similar positivity rates ranging from 23.27% to 26.96% were detected from 2016 to 2020 (Figure 2). However, the positivity rate in 2021 was comparably low (11.45%). With the exception of the 1st quarter of 2018 and the 2nd quarter of 2021, the positivity rates for the EA H1N1 subtype were the highest compared with the other subtypes or copositive subtypes during all quarters between 2016 and 2021. The highest positivity rate for the EA H1N1 subtype (43.22%) was detected in the 1st quarter of 2020, followed by the 4th quarter of 2018 (35.65%) and the 3rd quarter of 2016 (35.45%). Positivity rates for the EA H1N1 subtype ranged from 13.98% to 29.82% and were detected in most quarters. The lowest positivity rate of 5.76% was detected in the 2nd quarter of 2021, which was also lower than that of the pdm/09 H1N1 subtype (5.89%).

For the pdm/09 H1N1 subtype, lower positive rates were detected than for that of the EA H1N1 subtype, except for 2021. In 2016, 2017, 2019, and 2020, the positivity rates of the pdm/09 H1N1 subtype were less than 1/3 of those of the corresponding EA H1N1 subtype (Figure 2). In the 1st quarter of 2018, the highest positivity rate for the pdm/09 H1N1 subtype was 30.07%, which was more than 2-fold higher than that of the EA H1N1 subtype (13.98%) and contributed greatly to the overall positivity rate of 2018. Similar positivity rates ranging from 3.36% to 8.55% were detected in the other years. The highest positivity rates of the pdm/09 H1N1 subtype were detected in 2019 which contained the highest positivity rate of 22.1% in the 3rd quarter of 2019, followed by that of the 2nd quarter of 2019 (11.05%). Positivity rates of less than 10% were detected in all other quarters between 2016 and 2021. With the exception of the 3rd quarter of 2016, the 2nd−4th quarters of 2017, the 2nd quarter of 2018, and the 1st, 3rd, and 4th quarters of 2019, the H3N2 subtype was detected in all samples with positivity rates of less than 1%. The copositive samples for the EA H1N1and pdm/09 H1N1 subtypes were detected in all quarters with yearly positive rates of 13.88%, 19.22%, 12.38%, 18.79%, 11.35%, and 24.91% from 2016 to 2021, respectively. However, different positivity rates were observed between quarters, ranging from 0.07% in the 1st quarter of 2017 to 35.5% in the 2nd quarter of 2018. With the exception of the 1st quarter of 2017, positivity rates of 16.27%, 20.48%, and 27.14% were observed in the other three quarters of 2017. The highest positivity rate in the 2nd quarter of 2018 contributed greatly to the overall positivity rate in 2018. However, lower positivity rates (4.88%, 8.8%, and 10.76%) were detected in the 1st, 3^rd^, and 4th quarters of 2018. The highest copositivity rates to the EA H1N1 and pdm/09 H1N1 subtypes were detected in the 1st quarter (25.74%) of 2019, lower positivity rates were detected in the 3^rd^, 1^st^, and 4th quarters of 2019, with rates of 15.82%, 17.27%, and 18.09%, respectively. These results indicated that positivity rates may have little correlation with the quarters.

The copositivity rates for the EA H1N1 and H3N2 subtypes, pdm/09 H1N1 and H3N2 subtypes, and EA H1N1, pdm/09 H1N1, and H3N2 subtypes were lower than those of the EA H1N1 and pdm/09 H1N1 subtypes. Overall positivity rates of less than 1% were detected. Notably, the samples copositive for the EA H1N1 and H3N2 subtypes were more easily detected than the other two copositive types.

### 3.3. Feeding Stage Distribution of IAV-S-Positive Samples in China from 2016 to 2021

For individual pigs, different growth stages greatly influenced the susceptibility to IAV-S. In this study, five breeding stages including piglet pigs, nursery pigs, fattening pigs, gilts, and breeding pigs were divided. Breeding pigs were subdivided into sows and boars. The total seropositivity rates of piglets to IAV-S were the highest at 67.20%, followed by fattening pigs (55.28%), sows (48.67%), gilts (43.20%), nursing pigs (41.33%), and boars (41.02%). The dominant subtypes of IAV-S were variable based on different growth stages (Table 1).

For the EA H1N1 subtype, a positivity rate of 35.62% was detected in fattening pigs, which was much higher than that of the other groups (Table 1). The positivity rate of the EA H1N1 subtype in piglets was 30.35%; however, the EA H1N1 and pdm/09 H1N1 copositivity rate (30.00%) was much higher than that of other groups and contributed to the overall positivity rate. Similar positive rates (19.70%, 25.64%, and 23.43%) were also detected in nursery pigs, sows, and boars. The lowest positivity rate was detected in gilts. Lower positivity rates for the pdm/09 H1N1 subtype (4.64%–10.17%) were detected in groups compared to those of the EA H1N1 subtype. Fifteen of 15,121 sows were positive for the H3N2 subtype, and fewer than five samples that were positive for the H3N2 subtype were detected in other groups.

The copositivity rates for two or three subtypes were also greatly different. EA H1N1 and pdm/09 H1N1 copositive samples were the most common, with higher positivity rates than those of the pdm/09 H1N1 subtype in the same group. Copositivity rates of 19.26% and 12.48% for the EA H1N1 and pdm/09 H1N1 subtypes was observed in gilts and fattening pigs, respectively. Similar positivity rates were detected in nursery pigs, sows, and boars (13.92%, 12.53%, and 12.55%, respectively). The copositivity rates of the EA H1N1 and H3N2 subtypes were less than 0.1%, except in breeding pigs, which were similar with those of the copositivity rates of the pdm/09 H1N1 and H3N2 subtypes. Triple positive EA H1N1, pdm/09 H1N1, and H3N2 samples were also detected in piglets, reverse pigs, and breeding pigs with positive rates of 0.02%, 0.09%, and 0.07%, respectively. Six samples from 5,300 breeding boars were positive, for a positivity rate of 0.11%.

## 4. Discussion

EA H1N1 were first isolated from swine in Hong Kong in 1993 [13]. Since 2001, EA H1N1 IAV-S has become the dominant circulating virus in China [9, 14] and will preferentially bind to human-like receptors. Some of the viruses can be efficiently transmitted in ferrets through respiratory droplets [15], and preexisting immunity in humans may not be sufficient to overcome EAH1N1 infections [15, 16]. In fact, isolation of the EA H1N1 subtype has been reported from at least six cases in China [17–22].

In the serum samples from pig farm residents, antibodies specific to the EA H1N1 subtype were also detected with high positivity rates ranging from 10.4% to 11.7% [23–25]. Recently, a genotype 4 reassortant EA H1N1 virus was identified as the predominant strain in swine populations since 2016 and has acquired increased human infectivity [23]. Higher odds of antibody titers against EA H1N1 were detected in swine workers compared to the general population [26]. In this study, the serum samples positive for the EA H1N1 subtype were most abundant, with the highest positivity rate of 24.06% between 2016 and 2021. Those data further confirmed the predominance of the EA H1N1 subtype [23, 27–29]. The positivity rates based on regional distributions were variable and ranged from 3.7% (Central China) to 35.65% (North China) (Figure 1). The positivity rates were higher in South China from 2016 to 2019 and decreased in 2020, which may partially be due to the increased biosafety of pig farms against Africa swine fever disease. The high positivity rates to the EA H1N1 subtype indicated its predominance and increased in human infectivity, which indicated that it was a substantial threat to human health. Thus, more attention and funding toward the surveillance of the EA H1N1 subtype is urgently needed.

Since shortly after the emergence of pdm/09, the pdm/09 H1N1 subtype was spread to pigs throughout the world [30–32]. The reassortment between the pdm/09 H1N1 virus and endemic IAV-S has occurred frequently [8, 31], and IAV-S with surface genes of pdm/09 origin are rarely isolated [28, 31, 33, 34]. In this study, the positivity rate of the pdm/09 H1N1 subtype was 7.49%, which was lower than that of the EA H1N1 subtype. However, the copositivity rate to both EA H1N1 and pdm/09 H1N1 was 15.84%, which was higher than that of the pdm/09 H1N1 subtype. Those findings indicated that recombination between pdm/09 H1N1 and EA H1N1 may have occurred and was confirmed by virus sequencing in our studies and those of others [23, 28, 35].

Domestic pigs can independently facilitate the production of human pandemic strains with all gene segments of swine origin through rearrangement [12, 36]. The first human influenza pandemic of the twenty-first century was of swine origin and included the pdm/09 H1N1 virus, which is a quadruple reassortant containing genes from CS H1N1 IAV-S, human seasonal H3N2 influenza virus, North American avian influenza virus, and EA H1N1 IAV-S [36]. Serum samples from pig workers and residents were positive for pdm/09 H1N1 at a rate of 8.4% and 11.4%, respectively [37]. Moreover, almost 26% of residents in Shandong Province also carry antibodies against the pdm/09 H1N1 subtype [33, 34]. Positivity rates of 19% and 28.7% to the pdm/09 H1N1 subtype were also detected in serum samples from all types of outpatients and pediatric outpatients, respectively [38, 39]. Neutralizing activity against pdm/09 H1N1 was also detected in samples from humans [40]. Thus, all such results indicated that the pdm/09 H1N1 subtype is a threat to human and swine health, and more attention should be given to its prevalence.

H3N2 influenza viruses originated in humans and caused a major influenza epidemic in 1968 in southern China [11, 38]. Human H3N2 viruses were transmitted to pigs and reassorted with avian H9N2 and other viruses to produce new types of H3N2 viruses with pandemic potential [41]. The new types of H3N2 circulated in pigs long after the parent human virus had been replaced in the human population [11]. However, in this study, the positivity rate (0.06%) to the H3N2 subtype was quite low. Including copositive samples, a total of 102 of the 40,343 serum samples were positive for the H3N2 subtype, which indicated its low occurrence in pig herds. The detection of copositive serum samples indicated the high possibility of rearrangement of H3N2 with the EA H1N1 subtype or pdm/09 H1N1. Thus, new types of IAV-S are likely to appear repeatedly in the future.

Influenza viruses typically have obvious seasonality, which mostly occurs in late autumn, early spring, and cold winters, during large climate changes. Consistent with the findings of other groups [42], the results of this study also observed such seasonality. Notably, there were changes in seasonality after 2018 due to the outbreak of the African swine disease (ASF) [43]. Improvements to biosecurity issues in pig farms to combat ASF would also help to prevent IAV-S infections.

The results of this study showed that the positivity rates were correlated to some extent with the breeding stages of pigs. Serum samples from fattening pigs were detected with the highest positivity rates of 35.62%. In swine workers between 18 and 35 years of age, a 20.5% seropositivity rate to the G4 EA H1N1 subtype was detected, indicating that this was the predominant age group infected with that subtype [23]. Those results also suggested that young adults were more susceptible to the EA H1N1 subtype. The abundance of viral receptors may account for the high infectivity. The higher positivity rates of piglets to EA H1N1, EA H1N1, and pdm/09 H1N1 may also result from the presence of maternal antibodies, which can last for 2-4 months [44]. The results also showed that the group of longer-lived boars had the lowest positivity rates, which was due to the lower copositivity rates (Table 1). Thus, better biosecurity issues in the group of boars were implemented and helped to prevent viral infections.

## 5. Conclusions

Antibodies against the EA H1N1 and pdm/09 H1N1subtypes were easily detected in swine serum samples, which indicated that clinical infections were common from those viruses. The detection of copositive serum was indicative of the strong possibility of reassortment of EA H1N1 subtype with the pdm/09 H1N1or H3N2 subtypes. Thus, great attention should be paid to the appearance of new viral subtypes in the future. The findings of the present study further emphasized the need for detailed surveillance of IAV-S to understand its prevalence and provide a basis for its the prevention and control.

## Figures and Tables

**Table 1:** Serological survey of the feeding stages of IAV-S in the swine population in China, 2016-2021.

Pigs of different ages	EA H1N1	Pdm/09 H1N1	H3N2	EA H1N1 and pdm/09 H1N1	EA H1N1 and H3N2	Pdm/09 H1N1 and H3N2	EA H1N1, pdm/09 H1N1 and H3N2	Total
Piglets	1400/4613 (30.35)^a^	308/4613 (6.68)	4/4613 (0.09)	1384/4613 (30.00)	2/4613 (0.04)	1/4613 (0.02)	1/4613 (0.02)	3100/4613 (67.20)

Nursery pigs	800/4060 (19.70)	313/4060 (7.71)	0	565/4060 (13.92)	0	0	0	1678/4060 (41.33)

Fattening pigs	1507/4231 (35.62)	300/4231 (7.09)	0	528/4231 (12.48)	3/4231 (0.07)	1/4231 (0.02)	0	2339/4231 (55.28)

Gilts	1160/7018 (16.53)	500/7018 (7.12)	4/7018 (0.06)	1352/7018 (19.26)	6/7018 (0.09)	4/7018 (0.06)	6/7018 (0.09)	3032/7018 (43.20)

Sows	3877/15121 (25.64)	1538/15121 (10.17)	15/15121 (0.10)	1895/15121 (12.53)	16/15121 (0.11)	8/15121 (0.05)	10/15121 (0.07)	7359/15121 (48.67)

Boars	1242/5300 (23.43)	246/5300 (4.64)	1/5300 (0.02)	665/5300 (12.55)	10/5300 (0.19)	4/5300 (0.08)	6/5300 (0.11)	2174/5300 (41.02)

Total	9986/40343 (24.75)	3205/40343 (7.94)	24/40343 (0.06)	6389/40343 (15.84)	37/40343 (0.09)	18/40343 (0.04)	23/40343 (0.06)	19682/40343 (48.79)

^a^ *P*: positive samples; *n*: sample number; *R*: positive rate.

## Data Availability

The data used to support the findings of this study are included within the article.
